# Nanoscopic characterization of the water vapor-salt interfacial layer reveals a unique biphasic adsorption process

**DOI:** 10.1038/srep31688

**Published:** 2016-08-16

**Authors:** Liu Yang, Jianfeng He, Yi Shen, Xiaowei Li, Jielin Sun, Daniel M. Czajkowsky, Zhifeng Shao

**Affiliations:** 1 Shanghai Center for Systems Biomedicine, Shanghai Jiao Tong University, Shanghai, 200240, China; 2 School of Life Sciences, Tsinghua University, Beijing, 100084, China; 3 Bio-ID Center, School of Biomedical Engineering, Shanghai Jiao Tong University, Shanghai, 200240, China

## Abstract

Our quantitative understanding of water adsorption onto salt surfaces under ambient conditions is presently quite poor owing to the difficulties in directly characterizing this interfacial layer under these conditions. Here we determine the thickness of the interfacial layer on NaCl at different relative humidities (RH) based on a novel application of atomic force spectroscopy and capillary condensation theory. In particular, we take advantage of the microsecond-timescale of the capillary condensation process to directly resolve the magnitude of its contribution in the tip-sample interaction, from which the interfacial water thickness is determined. Further, to correlate this thickness with salt dissolution, we also measure surface conductance under similar conditions. We find that below 30% RH, there is essentially only the deposition of water molecules onto this surface, typical of conventional adsorption onto solid surfaces. However, above 30% RH, adsorption is simultaneous with the dissolution of ions, unlike conventional adsorption, leading to a rapid increase of surface conductance. Thus, water adsorption on NaCl is an unconventional biphasic process in which the interfacial layer not only exhibits quantitative differences in thickness but also qualitative differences in composition.

The adsorption of water molecules onto solid surfaces is a ubiquitous process under ambient conditions. As such, it plays important roles in many aspects of everyday life, from the aggregation of food particles, to the functioning of circuits, to the growth of clouds^1^,^2^,^3^. It is thus also a widely studied process, commonly described according to conventional adsorption models, such as the long-standing Langmuir-type isotherm^4^,^5^. While these models can also account for occurrences of multiple layers of molecules or interactions between molecules within a layer^6^, they nonetheless only describe adsorbed layers consisting of a single component with a substrate surface that remains unchanged during the process. However in practice, this is not always the case.

For example, water adsorption onto the table salt, sodium chloride (NaCl), at ambient conditions has long been known to be associated with the partial dissolution of the surface at humidities below deliquescence^7^,^8^. Solid NaCl is often studied as a model system to better understand the underlying collective physical mechanisms associated with salt dissolution^9^. It is also a major component of atmospheric aerosols, where processes occurring within and between interfacial water-salt layers are believed to be important for cloud formation and atmospheric chemical reactions^10^,^11^,^12^,^13^. Our understanding of these processes thus ultimately requires a precise understanding of the physical characteristics of this interfacial layer – its extent, composition, and the degree to which the water molecules are bulk-like – especially at different relative humidities.

As such, a range of techniques have been used to characterize adsorption/dissolution on this surface, including ellipsometry, infrared spectroscopy, quartz crystal microbalance, and scanning polarization force microscopy^14^,^15^,^16^,^17^. However, the results obtained using these methods with NaCl in particular, and with solid surfaces more generally, are not always in agreement and the interpretation of these data remains enigmatic^15^,^17^,^18^,^19^,^20^. For example, infrared spectroscopy infers the coverage of water on the surface based on the absorption spectrum assuming that the integrated cross sections at different humidities are the same, which has not been directly confirmed^15^. Clearly, a method to unambiguously characterize an aqueous interface is needed so as to provide a set of basic parameters of this important interfacial layer.

Here, we combine atomic force spectroscopy with the well-established theory of capillary condensation to provide a novel means of determining the thickness of the aqueous interfacial layer on NaCl(001) over a wide range of relative humidities. In addition, we also measured the surface conductance as a function of humidity as an indicator of salt dissolution into this interfacial layer. Overall, our results reveal that water adsorption on this surface is an unconventional, biphasic process, not explainable with existing theory, changing significantly in both thickness and composition at different humidities. In practical terms, these results indicate that atmospheric surface reactions within this layer that depend on interactions with water molecules will be profoundly dependent on the ambient relative humidity.

## Results

Interactions between an AFM tip and a given sample under ambient conditions have long been studied and are generally found to be dominated by electrostatic or van der Waals interactions and capillary condensation^21^,^22^,^23^,^24^,^25^,^26^. The latter, in this context, is the phenomenon in which water vapor condenses to form a fluid water bridge between the tip and sample, generating an attractive force between the two materials^27^,^28^,^29^. This previous work, though, did not explicitly separate the effects of capillary condensation from other possible sources of interaction and so the contribution of capillary condensation to a given tip-sample interaction was not clearly understood. However, while electrostatic or van der Waals interactions proceed essentially at rates set by the speed of light, capillary condensation occurs at a much slower rate, since it is a thermodynamic process associated with a free energy barrier^30^,^31^,^32^. In fact, over nanoscopic dimensions, Reido and collaborators found that at 34% RH, the characteristic rate of capillary condensation is 400 ± 50 μm/s, leading to an estimated nucleation time for one liquid layer of ~25 μs^31^. We thus reasoned that if the tip-sample approach speed was sufficiently fast such that the tip was within a few Å from the sample surface for much less than this nucleation time, condensation would not occur, and so any tip-sample interaction observed under these conditions would reflect only those owing to other sources of interaction. Hence, an understanding of the contribution of these other sources (at high tip-sample approach speeds) enables a direct measure of the contribution of capillary condensation in a given tip-sample interaction (at any approach speed). Further, since capillary condensation is also dependent on the amount of adsorbed water on the surfaces^33^, this measure of the extent of capillary condensation thus provides a means by which the thickness of the adsorbed water layers on the surfaces can be determined.

**Figure 1:**
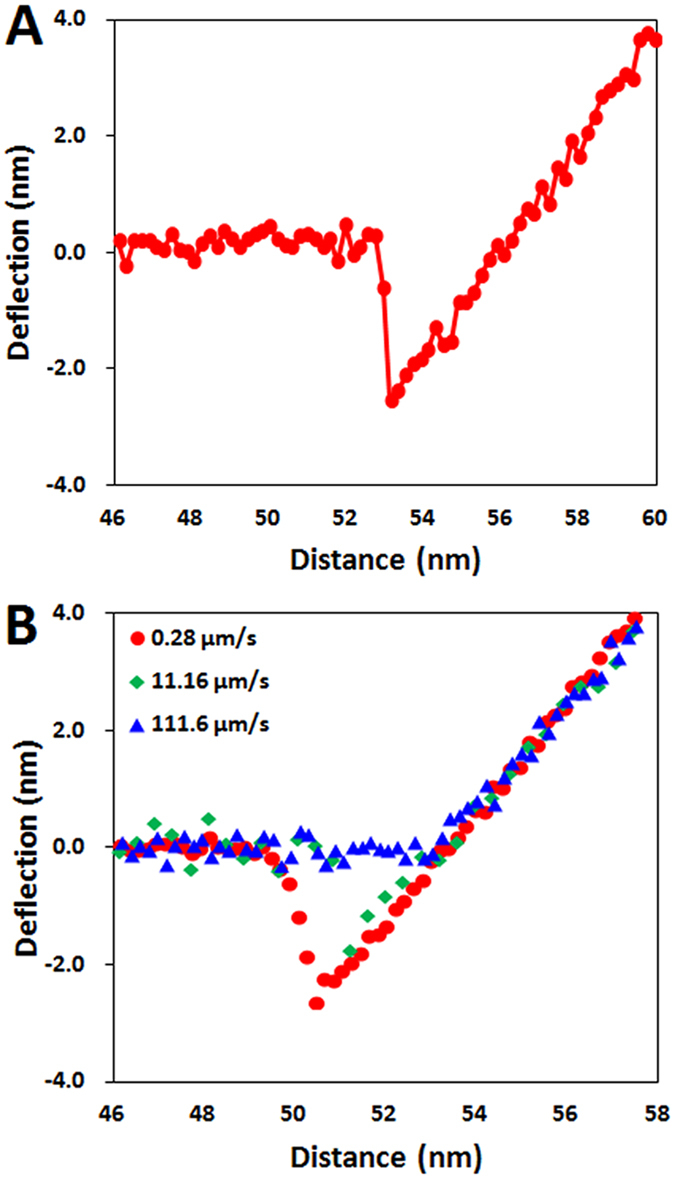
AFM force curves reveal a snap-in process that depends on tip approach speed. (**A**) At 37% RH, the 4.5 N/m AFM tip is found to snap-in to contact the NaCl(001) surface at a distance of ~2.5 nm. (**B**) The magnitude of the snap-in distance decreases as the tip approach speed increases.

We studied the interaction between a hydrophilic platinum (Pt)-coated tip with a freshly cleaved NaCl(001) surface under an exceptionally clean environment. Figure 1A shows that as the tip approaches the sample at a speed of 0.28 μm/s at 37% RH, it suddenly snaps-in to directly contact the salt surface at a distance of 2.5 nm from the sample surface. At this speed, the tip, if it did not snap-in, would have traversed this 2.5 nm over a time-frame of ~10^4^ μs, much longer than the expected time needed for capillary condensation. Thus, at least some of the attractive tip-sample interaction resulting in this snap-in could be owing to capillary condensation.

Figure 1B shows that at this same humidity, the magnitude of the snap-in distance decreases as the approach speed increases. In fact, at the highest approachable speeds, there is no detectable snap-in. At this maximal speed, the tip travels a distance of one water layer in ~2.7 μs, ~10-fold shorter than the expected time needed for capillary condensation. Thus, these results suggest not only that capillary condensation contributes to the attractive interaction associated with the snap-in, but also in fact that the observed snap-in is completely owing to capillary condensation.

**Figure 2:**
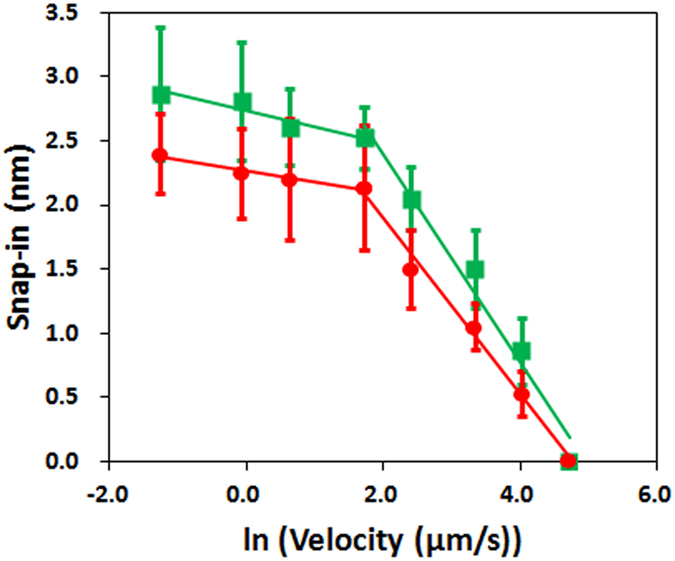
Speed dependence of the snap-in distance. There is little change in the snap-in distance at approach speeds smaller than ~7 μm/s. But above this value, the snap-in distance decreases with a logarithmic dependence. Red: 37% RH, and Green: 45% RH.

To further confirm the involvement of capillary condensation in this snap-in, we analyzed the speed dependence of the observed snap-in distance. Previous work has found that the height of the condensation bridge decreases with the logarithm of the speed at which the surfaces approach each other, and that the slope and intercept of this correlation provides a means by which the characteristic condensation speed, and likewise the nucleation time for a single layer of water molecules, can be determined^31^. As shown in Fig. 2, the snap-in distance indeed decreases with a logarithmic dependence of the tip approach speed. Further, the slope and intercept of this relationship are associated with a characteristic condensation speed of 120 μm/s and a single-layer nucleation time of 20 μs at 37% RH, similar to the nucleation time of nanoscopic capillary condensation in previous measurements^31^. We also observed a similar speed dependence and nucleation time in the snap-in at different relative humidities (Fig. 2). Finally, we note that, at a speed of 0.28 μm/s, the distance at which the tip “snaps-out” during tip retraction is larger than that at which it snaps-in (Supplementary Figure 1), consistent with a capillary condensation bridge that initially forms at equilibrium on tip approach being kinetically trapped during tip retraction owing to the relatively strong surface tension of water^34^. Overall, these results are consistent with the snap-in phenomenon in this system being completely owing to capillary condensation.

**Figure 3:**
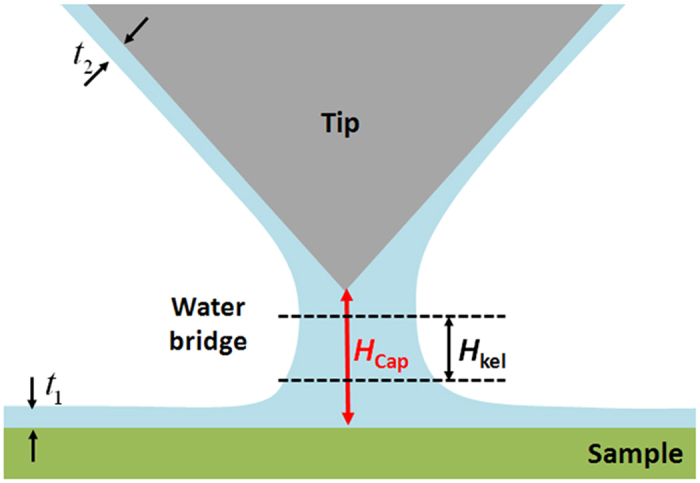
Schematic diagram of the capillary condensation between the AFM probe and the sample surface. Note that this distance also depends on the thickness of the water layers on the sample and tip (t_1_ and t_2_, respectively).

Previous studies of nanoscopic capillary condensation employed the well-known Kelvin-Laplace equation^28^,^29^,^35^. In the context of our AFM experiments, the height of the condensation bridge according to this equation is given by


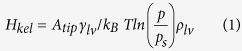


where *A*_*tip*_ is a constant that depends on the AFM tip shape, *γ*_*lv*_ is the surface tension of the liquid-vapor interface, *k*_*B*_ is Boltzmann’s constant, *T* is the temperature, *p* is the vapor pressure, *p*_*s*_ is the saturation vapor pressure and ρ_lv_ is the number density of the liquid. However, if there is also adsorbed water on either surface, the distance between the tip and sample surface will also depend on the thicknesses of the interfacial aqueous layers (Fig. 3 and Supplementary Figure 2)^33^. Hence, the total height of the capillary bridge including the contributions from the surface bound water is given by


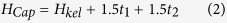


where *t*_*1*_ and *t*_*2*_ are the thicknesses of the interfacial layers on the sample and tip, respectively, which also accounts for the disjoining pressure^33^.

Since the snap-in phenomenon begins with the formation of the capillary condensation bridge and ends with the direct physical contact between the tip and sample, we equate the complete snap-in distance with *H*_*cap*_ and use Eqn 2 to determine the thickness of the interfacial layer on NaCl(001). This requires the knowledge of the thickness of the interfacial layer on the tip, which can be determined from measurements of the snap-in distance with a sample of the same material as the probe (Pt), in which case *t*_*1*_ = *t*_*2*_, once the snap-in measurement with this sample is also verified to be owing to capillary condensation (Supplementary Figures 3 and 4). We note that while previous studies have demonstrated the applicability of the Kelvin-Laplace equation at distances as low as ~1 nm 35, we experimentally validated this equation in our system by comparing the measured force gradients at different humidities with the theoretical values, finding excellent agreement (Supplementary Figures 5 and 6). We further note that, as shown in Supplementary Figure 5, measurements obtained with cantilevers that differ in spring constant overlap within experimental error, demonstrating that there are no significant measurement errors expected with results obtained with softer cantilevers.

**Figure 4:**
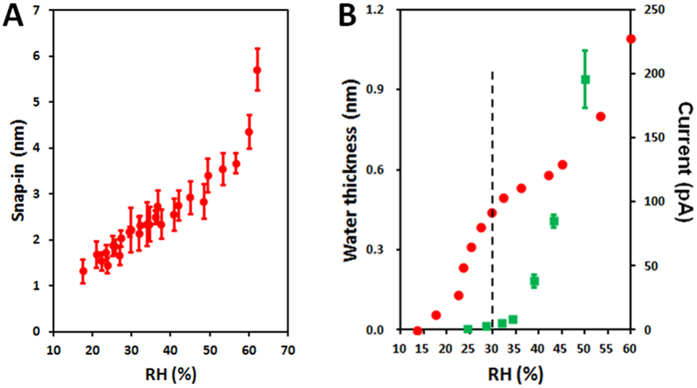
Humidity dependence of the interfacial water thickness and surface conductance on NaCl(001). (**A**) The snap-in distance over a wide range of relative humidities. (**B**) The interfacial layer thickness (red) and surface conductance (green) over a similar range of relative humidities. The black dotted line demarks the two regimes that exhibit different dependencies with humidity.

Figure 4A shows the dependence of the snap-in distance with the relative humidity (RH) and Fig. 4B shows the thickness of the adsorbed water on NaCl(001) calculated using Eqn 2 from the snap-in measurements. Supplementary Figure 7 shows the force curves on which these measurements are based, obtained with cantilevers with different spring constants. This isotherm exhibits two distinct regimes: below ~30% RH, there is a sigmoidal increase in layer thickness with RH up to a plateau value of ~4 Å, while above 30% RH, the thickness increases exponentially with RH, reaching ~11 Å at 60% RH (Fig. 4B). AFM images at both low and high RH remain featureless throughout the experiment (Supplementary Figure 8), verifying that the measurement itself did not lead to any changes to the sample surface.

As salt dissolution into the interfacial layer of NaCl(001) has been noted in previous work^7^,^8^, and would fundamentally change both the composition and thickness of this layer, we also examined the extent of this dissolution over a similar range of humidity by measuring the surface conductivity, taking this as an indicator of the presence of dissolved sodium or chloride ions (Fig. 4). Interestingly, we also observed two regimes: below 30% RH, there is no conductance while above 30% RH, there is a rapid, exponential increase in conductivity.

## Discussion

The adsorption of water on NaCl(100) under ambient conditions has long remained of great interest, both from a theoretical perspective as an important initial event during salt dissolution and from the more practical view of the significant role it is expected to play in many atmospheric reactions. Nonetheless, many basic physical properties of this process remain poorly understood. Here, we introduce a novel method by which the thickness of the interfacial water layer can be reproducibly measured under ambient conditions using AFM and capillary condensation theory. Together with the measurement of surface conductance, this work provides an unprecedented view of the changes in the dimensions and composition of this layer across a wide range of environmentally relevant relative humidities.

Strikingly, both measurements identify two qualitatively different regimes with the same ranges of humidity, and combined, reveal an unconventional, biphasic adsorption process of water on NaCl(001). Below 30% RH, and starting at 15% RH, there is only the adsorption of water molecules without the simultaneous dissolution of mobile ions from the surface, and this proceeds with RH until reaching a plateau of ~4 Å, the thickness of 1 to 2 layers of water molecules completely covering the surface. Such a description is qualitatively similar to conventional Langmuir type-1 adsorption process, where a single molecular species adsorbs until a single layer covers the surface^4^. Above 30% RH, adsorption of water molecules is simultaneous with the dissolution of surface ions, which hence also contributes to the measured thickness but more importantly, fundamentally changes the composition of this interfacial layer. Since all presently described types of conventional adsorption processes only involve a single molecular species within the layer, and especially do not include the possibility of the surface partially dissolving into the layer, new theory is clearly needed to describe this process.

We note that our results generally agree with a recent scanning tunneling microscopy study of the structure of water molecules on NaCl(001) at 77 K^36^. This work identified a novel bilayer ice structure on this surface that was present as local islands at low water coverages, which eventually covered the entire surface at higher surface densities. This layer was composed of only water molecules, with no dissolved surface ions, and exhibited a maximal thickness of ~3 Å. At high surface coverages (and after short exposure of the sample to higher temperatures), there was evidence for surface dissolution. Thus both the compositional differences at low and high water molecule surface densities and the maximal thickness of the pure water layer are consistent with our measurements, even though our data were obtained at room temperature. It is possible that this bilayer structure is only transiently present on the NaCl(001) surface at room temperature, together with a more disordered, but still purely water, organization of the molecules.

An interesting feature of the described process is the simultaneous dissolution of the surface ions only after more than 1 to 2 layers of water molecules adsorbed. It is clear that, during the first stage of water adsorption, the orientation and organization of water molecules is such that it is energetically favorable for the ions to remain within the crystal. Subsequent binding of water molecules to this first layer likely changes the orientation and organization of the molecules in this layer, as a consequence of the strong hydrogen-bonding ability of water molecules, as observed in other systems^19^,^37^. This altered organization of water molecules apparently favors the dissolved ions. It may be that the induced disorder of the water molecules in the first layer by the binding of water molecules in the second layer permits the rearrangements in the water organization necessary to favorably restructure around the dissolved ions.

Finally, as mentioned above, our results are also of practical significance, as there are many environmentally important chemical reactions that are known to occur within the surface of airborne NaCl crystals^13^. Since both the thickness, and more importantly the composition and intermolecular organization, of the interfacial aqueous layer will change at different humidities, it is expected that the rates of chemical reactions and the types of reactions that are possible within this interfacial layer will also be sensitively dependent on the humidity, particular around 30% RH. Indeed, with so significant changes in the hydrogen-bonding network of the water molecules within this layer with RH, there may be chemical reactions that occur within this layer at one RH that do not occur at other RH or in bulk water.

In conclusion, the present work represents a significant advance in our understanding of water adsorption on NaCl and, more generally, our ability to measure the thickness of the interfacial water layer on a solid surface under ambient conditions. As the adsorption of water molecules onto solid materials is a ubiquitous process that influences the functioning of many materials, we anticipate that this method will find wide application in more detailed studies of the functional consequences of this common but quite poorly understood process.

## Methods

### Experimental materials and protocol

The NaCl sample (SPI Supplies Division Structure Probe, Inc.) was cleaved and all AFM measurements were performed in an air-tight glove box thoroughly purged with nitrogen gas. With extended purging, a low relative humidity (~5% RH) could be reliably achieved. Exposure to a small solution of water enabled well-controlled adjustments to the humidity within the glove box. The force curves were acquired using a Nanoscope IVa-Multimode AFM (Veeco-Digital Instruments, Santa Barbara, CA) with an E scanner and NSC35 Ti/Pt probes (R ~ 40 nm, MikroMasch, ISB Ltd, Bulgaria) at ~21 °C. For the experiments with Pt-coated tips, we note that, as described in ref. 27, for surfaces of curvature greater than ~30 nm, there is no significant influence of surface curvature on water adsorption even at humidities as low as 10% RH. The spring constants of the cantilevers were determined from measurements of the resonance frequency and were within 20% of the manufacturer’s suggested values. As in previous work^38^,^39^, we monitored the adhesion force to identify any changes in the tip size during the experiments. Throughout these experiments, the adhesion force was unchanged, consistent with a constant tip size and shape during these experiments. The average and standard deviation of each “snap-in” distance was calculated from more than 200 AFM force-distance curves at each humidity. In the speed-dependence experiments, results were gathered by a data acquisition card rather than the Nanoscope software to increase the sampling rate. We note that the response time of the cantilever (half of the resonance period) used to obtain Fig. 2 is 3 μs, more than two orders of magnitude smaller than the residence time (~448 μs) of an AFM tip at a distance of ~2.5 nm from the surface, with an approaching speed of 5.6 μm/s (if the tip did not snap-in to contact the surface). Further, the residence time at the fastest speeds examined in this experiment (111.6 μm/s) over this ~2.5 nm distance is still ~22 μs, over seven-fold larger than the bandwidth of the cantilever. Thus, these experiments were not limited by the mechanical bandwidth of the cantilever^40^. Further, we note that, since the detection electronics of our instrument has a usable bandwidth of ~2 MHz^41^, our measurements are also not limited by the instrumentation electronics.

The SEM image in the inset to Supplementary Figure 2b was acquired using a Zeiss LEO 1530 VP FE-SEM.

The surface conductivity was measured using an Axon Axopatch 200 B patch clamp amplifier (Molecular Devices LLC, Sunnyvale, CA) at ~21 °C inside the aforementioned glove box after purging with nitrogen gas overnight. The sodium chloride crystal was cleaved twice inside the glove box to produce a ~1 mm thick sample with two new surfaces on which the surface current was measured. Indicated surface conductivities reflect triplicate measurements of two independent samples. The electrodes (two alligator clips) connected to the patch-clamp amplifier were attached on opposing ends of the same surface of the crystal. The voltage is thus applied across the crystal surface, thereby enabling measurement of the surface current.

### Theoretical considerations

We used the classical Kelvin equation to calculate the profile of the condensate water bridge. By considering thermodynamic equilibrium (Eqn 3) and geometry equilibrium (Eqn 4), we can obtain the exact shape (height and curvature) of the water bridge (Supplementary Figure 2a).


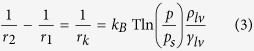



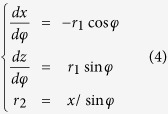


where, *r*_*1*_, *r*_*2*_ are the bridge curvature radii, *r*_*k*_ is the Kelvin radius and *φ* is the bridge tilting angle.

By considering this tip geometry, we obtain the following relation:


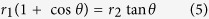


Here, *θ* is the horizontal angle of the tip, which is determined by the tip radius, R (see Supplementary Figure 2b). Combining Eqns 2 and 5, we have:


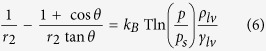


From geometry (see Supplementary Figure 2b), we have the height of the condensate water bridge, H*kel*, is given by:


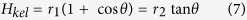


Thus, for the Pt-coated tip used in our experiments, we have, combining Eqns 6 and 7:


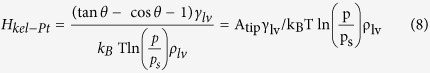


where A_tip_ = tan *θ*−cos *θ*−1.

We note though that we also investigated spherical and parabolic tip geometries, and these did not significantly change the height of the calculated condensation bridge (data not shown).

After the profile is obtained, the capillary force gradient can be calculated based the Laplace equation, where x_0_ is the horizontal radius of the water meniscus that contacts the AFM tip:


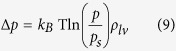



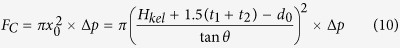



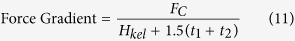


### Characteristic speed for capillary condensation

To obtain the characteristic speed for capillary condensation, *v*_*A*_, from the data presented in Fig. 2, we obtained an expression for the height of the (non-equilibrium) capillary condensation bridge, following ref. 31. In short, the nucleation energy barrier associated with capillary condensation is given by


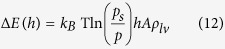


where *h* is the height of the water bridge, and *A* is the cross-sectional area of the bridge. The time to nucleate a bridge of height *h* is given by


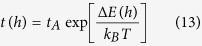


where *t*_*A*_ is the condensation time of a liquid monolayer. From *t(h)*, the non-equilibrium height that a liquid bridge forms at a certain time *t* is thus given by


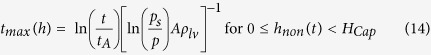


or, at a given speed *v*, we finally obtain


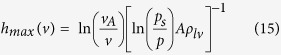


where *v*_*A*_ is the characteristic speed for capillary condensation. We note that the equilibrium condensation bridge height is determined from the Kelvin-Laplace equation (*H*_*Cap*_), and that the snap-in distance changes from independent of the approach speed to this logarithmic dependence at the critical tip speed, *v*_*crit*_, where *h(v*_*crit*_) = *H*_*Cap*_.

### Measurement of the force gradient

The snap-in effect occurs when the force gradient of the tip-sample interaction exceeds the spring constant of the cantilever. Thus at force gradients smaller than the spring constant of a given cantilever, there is no snap-in and at force gradients above the spring constant, there is a snap-in. We found that, for many cantilevers, there was no snap-in at low humidity and that there was a specific humidity beyond which there was a snap in (Supplementary Figure 5a). This specific humidity was different for cantilevers with different spring constants, namely larger for stiffer cantilevers (Supplementary Figure 5a). We thus used the spring constant of the cantilever as a measure of the force gradient at the relative humidity at which the snap-in was first observed (Supplementary Figure 5a). This provided one set of direct measurements of the force gradient of the condensation bridge. In addition, close inspection of the force curves at the lower humidities where there is no snap-in revealed that the cantilever deflects down on approach at a distance of a few nanometers from contact with the surface (Supplementary Figure 5b). We thus used the slope of this slower deflection as a measure of the force gradient at those humidities where a snap-in was not observed.

## Additional Information

**How to cite this article**: Yang, L. *et al*. Nanoscopic characterization of the water vapor-salt interfacial layer reveals a unique biphasic adsorption process. *Sci. Rep.* **6**, 31688; doi: 10.1038/srep31688 (2016).

## Supplementary Material

Supplementary Information
