# Supplementary material for: Nanoscopic characterization of the water vapor-salt interfacial layer reveals a unique biphasic adsorption process
Source: Sci Rep. 2016 Aug 16;6:31688. doi: 10.1038/srep31688 (PMC4985642; doi:10.1038/srep31688)
Supplement: Supplementary Information [file srep31688-s1.pdf]

Supplementary Information for

Nanoscopic characterization of the water vapor-salt interfacial layer reveals a unique biphasic adsorption process

Liu Yang, Jianfeng He, Yi Shen, Xiaowei Li, Jielin Sun, Daniel M. Czajkowsky & Zhifeng Shao

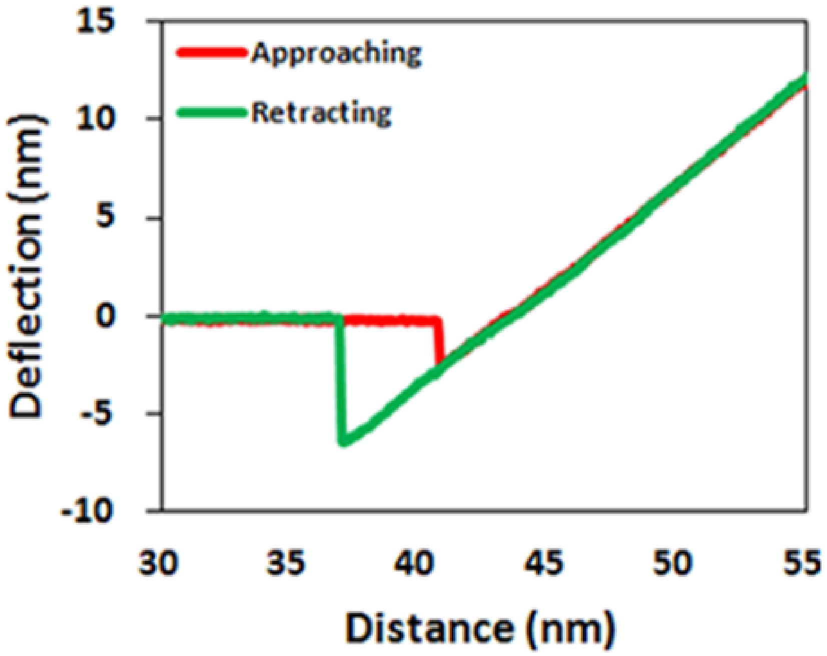

Supplementary Figure 1. AFM cantilever deflection on approach to and during the retraction from the NaCl(001) surface. The snap-out distance during retraction is greater than the snap-in distance on approach, as expected for capillary condensation. The data were obtained with a 4.5 N/m cantilever at 37 %RH.

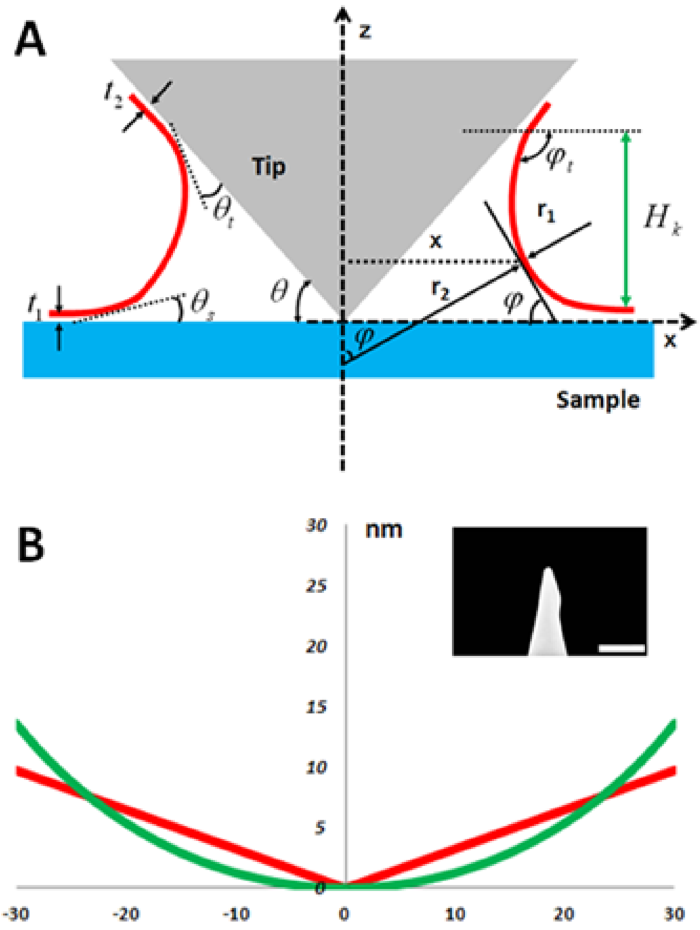

Supplementary Figure 2. Schematic diagram of the condensation bridge showing the key parameters of the Kelvin equation. (a) The r_1, r_2 are the bridge curvature radii, φ is the bridge tilting angle, θ_s is the water contact angle of the sample surface, θ_t is the water contact angle of the tip surface, θ is the horizontal angle of the tip, t_1 and t_2 are the surface water thicknesses, and H_k is the height of the water bridge without the surface water influence. (b) The ultimate tip apex in these experiments is essentially spherical with a radius of ~40 nm (green), which was approximated as a cone (red) in our calculations. Inset: An SEM image of a typical 4.5 N/m tip used in these experiments. Scale bar: 500 nm.

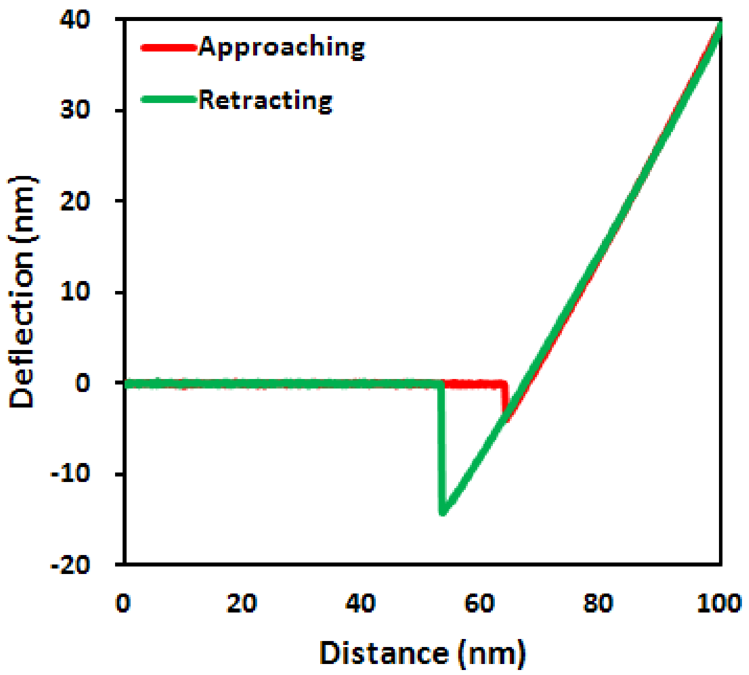

Supplementary Figure 3. AFM cantilever deflection during approach or retraction from a Pt surface. As observed with NaCl(001), the snap-out distance during retraction is greater than the snap-in distance on approach, as expected for capillary condensation.

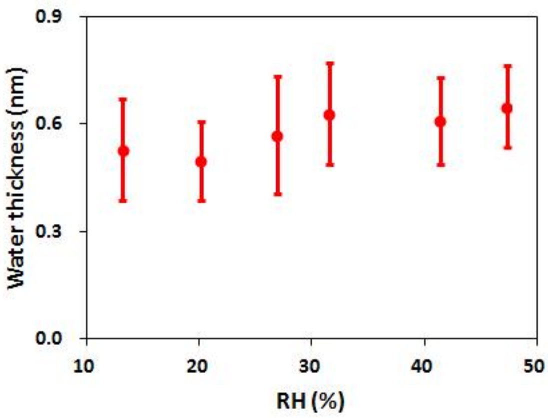

Supplementary Figure 4. The thickness of the interfacial layer on the Pt surface.

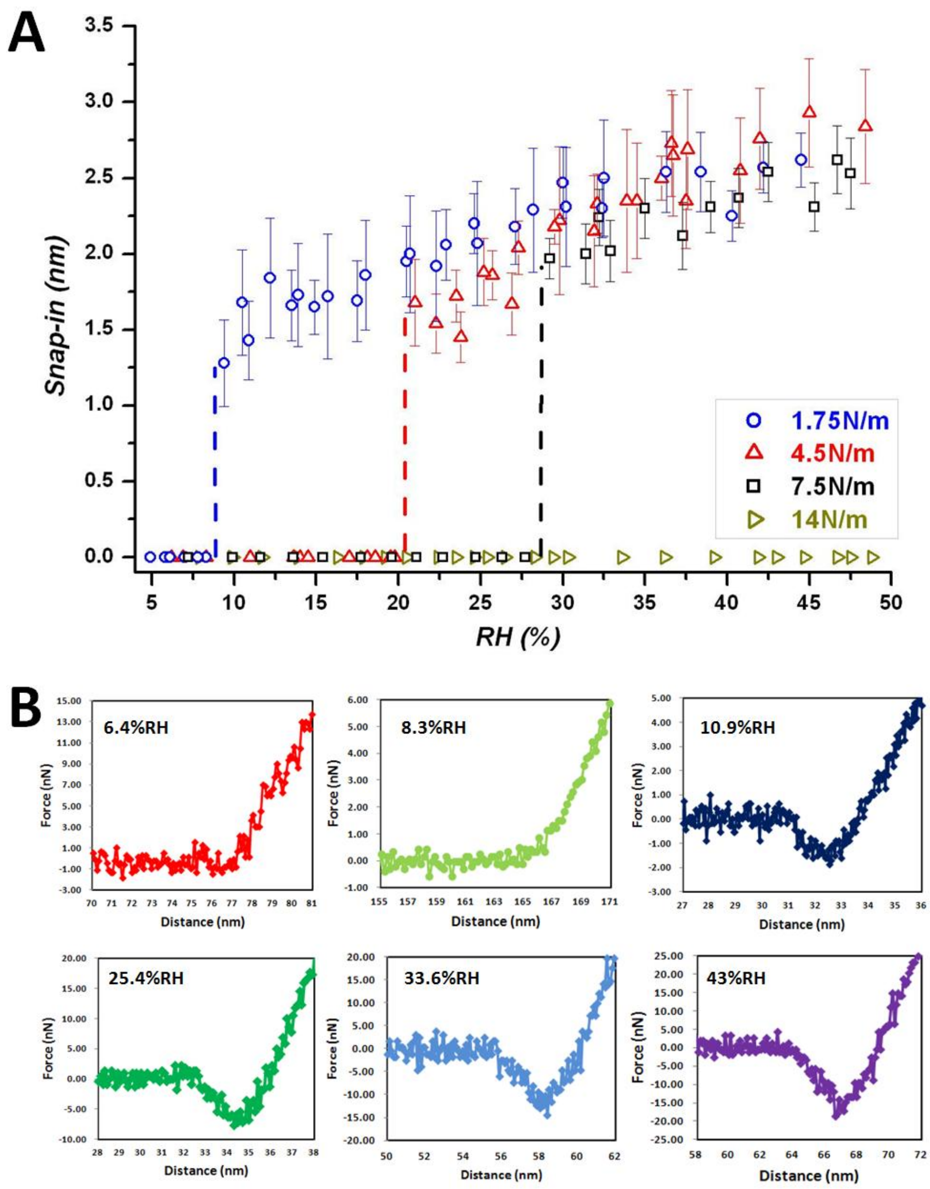

Supplementary Figure 5. (a) At lower humidities, there is no cantilever snap-in since the tip-sample force gradient is lower than the spring constant of the cantilever. However, this force gradient increases with humidity and when it exceeds the spring constant of the cantilever, the cantilever snaps in. Thus, the spring constant of the cantilever is a measure of the force gradient at the humidity at which the snap-in effect first emerges. (b) Force curves from a NaCl(001) surface obtained with a 14 N/m cantilever.

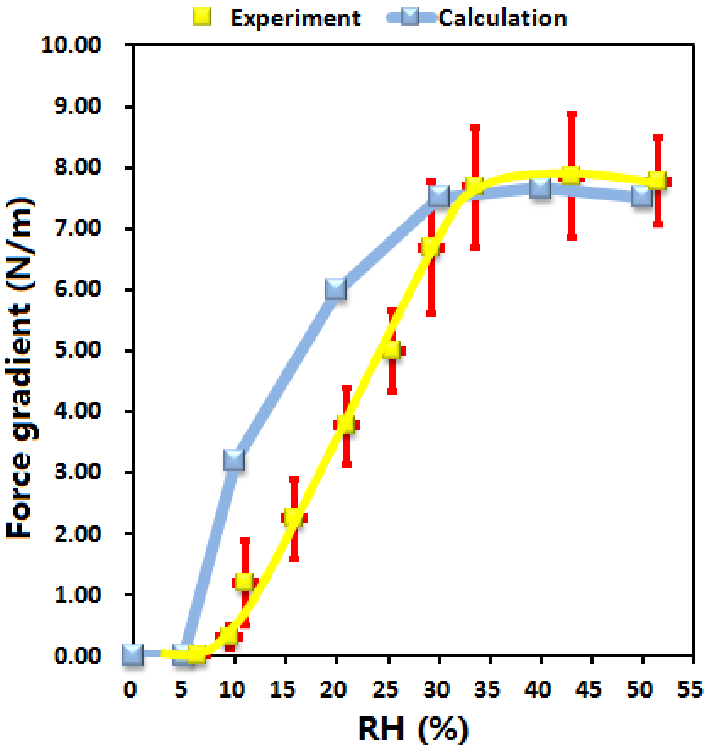

Supplementary Figure 6. Comparison of the force gradients obtained in this experimental way with that expected from the Kelvin equation (Equation 3-7).

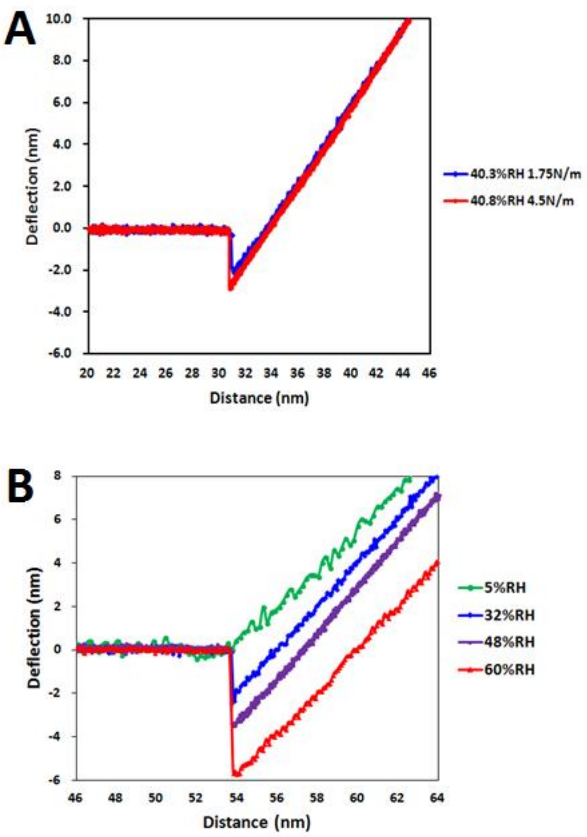

Supplementary Figure 7. The AFM force curves on which the measurements are based. (a) The force curves obtained with cantilevers with different spring constants at the same humidity are the same. (b) The force curves obtained at different humidities with different cantilevers.

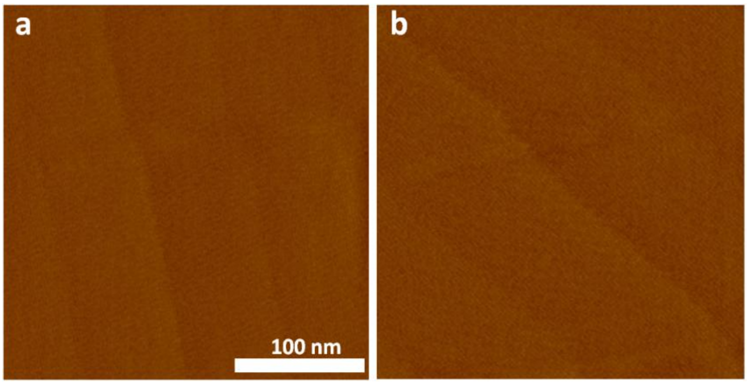

Supplementary Figure 8. AFM images at both low and high RH remain featureless throughout the experiment, verifying that the measurement itself did not lead to any changes to the sample surface. (a) 13.5%RH and (b) 37.6%RH.
